# Does a relationship still exist between gastroesophageal reflux and *Helicobacter pylori* in patients with reflux symptoms?

**DOI:** 10.1186/1477-7819-12-375

**Published:** 2014-12-06

**Authors:** Michele Grande, Giorgio Lisi, Flavio De Sanctis, Simona Grande, Alessia Esser, Michela Campanelli, Valerio Balassone, Giovanni Milito, Massimo Villa

**Affiliations:** Department of Experimental Science and Surgery, Policlinico “Tor Vergata”, Viale Oxford, 81, 00133 Rome, Italy; Department of Surgery, University Hospital Tor Vergata, Viale Oxford, 81, 00133 Rome, Italy

**Keywords:** Helicobacter pylori, RE, GERD, Esophageal manometry, Endoscopic procedure

## Abstract

**Background:**

The nature of the relationship between *Helicobacter pylori* and reflux esophagitis (RE) is not fully understood. In addition, the effect of *H. pylori* eradication on RE and gastroesophageal reflux disease (GERD) is unclear. This study was designed to investigate the relationship between *H. pylori* infection and the grade of GERD in patients with reflux symptoms.

**Methods:**

Between January 2010 and July 2013, 184 consecutive patients with daily reflux symptoms for at least one year were evaluated at the ambulatory for functional esophageal disease, Tor Vergata University Hospital, Rome, Italy. All patients underwent a pretreatment evaluation, which included anamnesis, clinical examination, Esophagogastroduodenoscopy (EGDS) with biopsy, esophageal manometry and 24-hour pH-metry. All statistical elaborations were obtained using Statigraphies 5 plus for Window XP.

**Results:**

There was no statistical difference regarding Lower Esophageal Sphincter (LES) pressure between patients who were *H. pylori*-positive and *H. Pylori*-negative (19.2 ± 9.5 (range: 3.7 to 46.2) and 19.7 ± 11.0 (range: 2.6 to 61), respectively). Further, no significant difference was evidenced in esophageal wave length (mean value: 3.1 seconds in *H. pylori*-negative patients versus 3.2 seconds in *H. pylori*-positive patients) or in esophageal wave height (mean value: 72.2 ± 39.3 in *H. pylori*-negative patients versus 67.7 ± 28.4 in *H. pylori*-positive patients). We observed that hiatal hernia (*P* = 0.01), LES opening (*P* = 0.05), esophageal wave length (*P* = 0.01) and pathological reflux number (*P* = 0.05) were significantly related to the presence of esophagitis. However, *H. pylori* infection was not significantly related to the presence of reflux esophagitis.

**Conclusions:**

Our clinical, endoscopic, manometric and pH-metric data shows no significant role of *H. pylori* infection in the development of GERD or in the pathogenesis of reflux esophagitis. However, current data do not provide sufficient evidence to define this relationship and further prospective large studies are needed.

## Background

*Helicobacter pylori* (HP) infection plays a major role in the pathogenesis of peptic ulcer disease, chronic gastritis, gastric mucosa-associated lymphoid tissue lymphoma (MALToma) and the development of gastric cancer. However, its role in reflux-induced esophageal injury (reflux esophagitis (RE)) and gastroesophageal reflux disease without esophagitis (GERD) is not fully understood. In addition, the effect of *H. pylori* eradication on RE or GERD is unclear. Several reports have shown an association of *H. pylori* eradication with the development of RE or GERD symptoms [1–3]. However, contradictory results have been reported, and sometimes a beneficial effect of *H. pylori* eradication on GERD has been observed [4–6].

Despite the results of these studies, it is conceivable that *H. pylori* could contribute to esophagitis in GERD through any of at least three potential mechanisms: 1) *H. pylori* infection might cause a predisposition to GERD by increasing gastric acid secretion, 2) *H. pylori* might cause esophageal damage by directly infecting the gastric-type columnar epithelium that can line the distal esophagus normally or as part of Barrett's esophagus or 3) *H. pylori* might cause esophageal damage indirectly through the action of noxious substances that are secreted by the organism into the refluxed gastric juice [7].

Colonization of gastric mucosa by *H. pylori* may result in hypochlorhydria in patients with diffuse gastritis and gastric atrophy and who seem to be at less risk of developing GERD [8]. Therefore, association between *H. pylori* infection and development of either hypochlorhydria or hyperacid secretion depends on the inflammatory response of the gastric mucosa. Thus, the effect of *H. pylori* infection on the development of GERD is contradictory and is an intricate relationship [8]. This study was designed to investigate the relationship between *H. pylori* infection and the grade of GERD in patients with reflux symptoms.

## Methods

Between January 2010 and July 2013, 184 consecutive patients with daily reflux symptoms for at least one year were evaluated at the ambulatory for functional esophageal disease, Tor Vergata University Hospital, Rome, Italy, and were included in this prospective study. The study had been approved by the Institutional Committee of the Tor Vergata University of Rome.

We classified the symptoms of the patients as did Patti *et al*. [9]; a patient-estimated scale ranging from 0 (no symptom) to 4 (disabling symptom). Swallowing status was graded as follows: excellent (no dysphagia), good (occasional dysphagia), fair (frequent dysphagia requiring dietary adjustments) or poor (severe dysphagia preventing the ingestion of solid food).

Exclusion criteria were: previous therapy to eradicate *H. pylori*, concomitant assumption of aspirin and non-steroidal antiinflammatory drugs or previous surgical procedures on the digestive tract. All patients underwent pretreatment evaluation, which included anamnesis, clinical examination, EGDS with biopsy, esophageal manometry and 24 hours pH-metry. Symptoms (heartburn, pain and regurgitation) were assessed by patients' visits. Outpatient manometry and pH studies were performed using a conventional protocol.

The study has not evaluated the degree of GERD as we are only interested in the correlation between RE and GERD. Kusano *et al*. [10] have shown that the Frequency Scale for Symptoms of GERD (FSSG) is useful only to evaluate therapeutic efficacy in GERD patients during the follow-up period without repeated endoscopy.

### Esophageal manometry

All subjects presented for a manometry after fasting for a minimum of eight hours. Medications known to inhibit or affect esophageal motility (nitrates, metoclopramide hydrochloride or calcium channel-blocking agents) were discontinued five days before manometry or 24-hour pH monitoring. A six-way perfusion water catheter (three radial and three spaced at 1-cm intervals) was used for all studies. The catheter was calibrated from 0 to 50 mmHg immediately before use (Medtronic Polygram Net™ version 4.01. Tonsbakken, Denmark).

An esophageal manometry was performed in order to define the position, extension, pressure of Lower Esophageal Sphincter (LES) (resting pressure and relaxation), amplitude, duration and velocity of esophageal peristaltic. Esophageal motility and gastroesophageal junction coordination were evaluated using 5 ml of room temperature water in a sequence of 10 swallows. Double swallows and swallows including pressure artefacts associated with coughing or belching were not counted in the 10 swallows.

### 24-hour esophageal pH monitoring

During the study the patients consumed an unrestricted diet and took no medications that could interfere with the results. Esophageal pH monitoring was conducted using an antimony pH catheter with external or internal reference (AccuView pH, Sierra Scientific Instruments. Yoqneam, Israel). The sensor was positioned 5 cm above the LES. Continuous pH recording was performed for 24 hours.

The DeMeester score [11] was used to calculate the following distal pH variables: percentage of total time that pH was less than 4, percentage of supine time that pH was less than 4, percentage of upright time that pH was less than 4, longest reflux event, number of reflux events longer than five minutes, and number of reflux episodes in 24 hours. A DeMeester score of greater than 14.72 was considered abnormal.

In patients with an abnormal reflux score (reference reflux score: 14.7), tracings were analyzed to distinguish between false reflux (due to stasis and fermentation) and true reflux [8]. When the clinical picture was ambiguous (a patient with good response to Proton Pump Inhibitors (PPIs) and esophagitis, or a normal but borderline reflux score), the pH monitoring study was repeated after three weeks.

### Endoscopic procedure

All study subjects underwent upper gastrointestinal endoscopic examinations. The endoscopic findings of RE in the lower esophagus were classified according to the Savary-Miller classification [12], based on the longest length of the mucosal break and confluence of erosions. Esophagitis was graded by endoscopy: grade 0, no lesions; grade 1, esophagitis was defined as the presence of single or isolated erosion on one mucosal fold; grade 2, non-circumferential erosions on more than one mucosal fold with or without confluence; grade 3, circumferential erosions that may be confluent; grade 4, ulcer, stricture, or esophageal shortening and Barrett's epithelium.

A hiatal hernia was endoscopically defined as a distance from the esophagogastric junction to the diaphragmatic impingement of more than 1 cm. The esophagogastric junction was defined as the proximal margin of the gastric mucosal fold [3]. Barrett's esophagus has been defined as the presence of squamo-columnar metaplasia localized at least 3 cm above the esophagus-gastric junction; two to three samples of the lower esophagus (last 3 cm) were obtained [12]. An endoscopic biopsy both of the gastric body and the antrum was performed in order to diagnose *H. pylori* infection and to obtain histological evaluation of the mucosa. *H. pylori* infection was diagnosed by the color-coded biopsy test (CCBT); we did not choose the stool antigen test (SAT) because CCBT is more feasible, faster to perform and executable at the time of the endoscopic procedure. The specimens were preserved in formaldehyde solution (10%) for histopathologic evaluation.

### Statistical analysis

All statistical elaborations were obtained by using Statigraphies 5 plus for Window XP (Statsoft; Tulsa, Oklahoma, United States). Results are expressed as mean values and standard deviation (SD). Quantitative variables between the two groups (*H. pylori*-positive and *H. pylori*-negative patients) were compared using the Student's t-test; qualitative parameters were compared between the two groups using chi-squared test. Results were considered statistically significant at *P* <0.05.

## Results

The present study included 184 patients (73 males and 111 females) with a mean age of 51.5 ± 15.2 years (range: 23 to 89). The baseline characteristics of the two groups are shown in Table 1.

**Table 1:** **Demographics characteristics of the 184 patients according to the presence or absence of** ***H. pylori*** **infection**

Demographic variables	***H. pylori*** -negative patients, n (%)	***H. pylori*** -positive patients, n (%)	Total patients, n (%)
**Number of patients**	99 (53.8)	85 (46.2)	184 (100)
**Men**	41 (41.4)	32 (37.6)	73 (39.1)
**Women**	58 (58.6)	53 (62.3)	111(51.6)
**Age range, years (median)**	24 to 74 (48.87)	17 to 73 (50.1)	17 to 74 (49.72)

All patients suffered from daily reflux symptoms for at least one year, there was no statistical difference regarding the severity of symptoms and symptoms score as given by the patients between the two groups (Table 2).

**Table 2:** Clinical parameters and symptoms score* of 184 patients with gastroesophageal reflux disease

Symptoms	***H. pylori*** -positive	***H. pylori*** -negative	***P*** value
Patients	85/184	99/184	
Regurgitation (%)	36 (42.3%)	61 (61.6%)	NS
(Score 0-4)	(1.56 ± 0.81)	(1.59 ± 0.11)	
Dysphagia (%)	7 (8.2%)	21 (21.2%)	NS
(Score 0-4)	(1.86 ± 0.26)	(1.57 ± 0.15)	
Heartburn (%)	39 (45.9%)	61 (61.6%)	NS
(Score 0-4)	(1.82 ± 0.10)	(1.90 ± 0.09)	
Epigastric pain (%)	14 (16.5%)	17 (17.2%)	NS
(Score 0-4)	(1.50 ± 0.17)	(1.47 ± 0.17)	
Thoracic pain (%)	29 (34.1%)	45 (45.4%)	NS
(Score 0-4)	(1.34 ± 0.10)	(1.89 ± 0.44)	
Dyspepsia (%)	12 (14.1%)	17 (17.2%)	NS
(Score 0-4)	(1.75 ± 0.18)	(1.71 ± 0.17)	
Other symptoms (%)	14 (16.5%)	21 (21.2%)	NS

*Data are given as mean ± standard error. Scores range from 0 (best) to 4 (worse). NS, Not Significant.

*H. pylori* infection was diagnosed in 85 patients (46.2%; 32 males and 53 females), while 99 patients (53.8%; 41 males and 58 females) were *H. pylori*-negative. Patients with and without *H. pylori* infection were statistically compared. There were no significant differences between the two groups regarding age, gender and presentation of symptoms. Hiatal hernia was found in 122 out of 184 patients (66.3%); 31 of which were *H. pylori*-positive (25.4%) and 91 were *H. pylori*-negative (74.6%), this data was statistically significant (*P* <0.0001). Out of these 122 patients with hiatal hernia, 40 (32.8%) suffered from RE, and in this group *H. pylori*-infection was diagnosed in 10 patients (25%), therefore in 82 patients (67.2%) with GERD and no RE, *H. pylori*-infection was present in 21 cases (25.6%). The incidence of hiatal hernia between the group with RE (40 patients) and the group without RE (82 patients) was significant (*P* <0.0001).

According to the Savary-Miller classification [9], 132 patients (71.7%) were graded 0 and had GERD only, and in 52 out of 184 patients (28.3%) reflux esophagitis was evidenced, of which 18 patients were graded 1 to 3 (four *H. pylori*-positive patients and 14 *H. pylori*-negative) and 34 patients were graded 4 (11 *H. pylori*-positive patients and 23 *H. pylori*-negative); two of these cases were found to have Barrett’s esophagus, and both were *H. pylori*-negative. Impairment of esophageal motility and presence of waves dropped and not forwarded was detected at manometry in 140 patients out of 184 (76%). *H. pylori* was present in 33 of these (23.6%), while 107 were *H. pylori*-negative (76.4%).

There was no statistical difference regarding LES pressure between patients who were *H. pylori*-positive and *H. pylori*-negative (19.2 ± 9.5 (range: 3.7 to 46.2) and 19.7 ± 11.0 (range: 2.6 to 61), respectively). Further, significant difference was not evidenced in either esophageal wave length (mean value: 3.1 seconds in *H. pylori*-negative patients versus 3.2 seconds in *H. pylori*-positive) nor in esophageal wave height (mean value: 72.2 ± 39.3 in *H. pylori*-negative patients versus 67.7 ± 28.4 in *H. pylori*-positive), (Table 3).

**Table 3:** **Manometric data of 140 patients with esophageal impairment motility**

Parameters	***H. pylori***-positive	***H. pylori*** -negative	***P*** value
Patients	33	107	
LES pressure (mmHg)	19,2 ± 9,5	19,7 ± 11,0	NS
v.n. 14.3-34.5 mmHg			
Esophageal wave length (seconds)	3.2	3.1	NS
v.n. 2.9-5.1 (mmHg)			
Esophageal wave height (mmHg)	67.7 ± 28,4	72.2 ± 39,3	NS
v.n. 33-91 (mmHg)			

NS, Not Significant.

The pH-metric parameters, that is, reflux episodes, pathological reflux episodes (refluxes with pH <4 that last over five minutes) and extent of esophageal acid exposure, were similar in both groups (Table 4).

**Table 4:** **pH-metric data of 95 patients**

Parameters	***H. pylori*** -positive	***H. pylori*** -negative	***P***
**Patients**	22	73	
**Reflux episodes**	113.9 ± 147.8	135.1 ± 129	NS
**Pathological reflux episodes**	3.3 ± 6.7	2.4 ± 4.3	NS
**Esophageal acid exposure (minutes)**	117 ± 195.2	109.3 ± 205.5	NS
**DeMeester score**	35.9 ± 56.7	33.3 ± 48.4	NS

NS, Not Significant

Out of 146 patients, 95 (51.6%) had pathological values on the DeMeester scale; 22 patients were *H. pylori*-positive (23.1%) and 73 were *H. pylori*-negative (76.8%). The mean value of the DeMeester score was 35.9 ± 56.7 in *H. pylori*-positive patients versus 33.3 ± 48 in *H. pylori*-negative, and this difference was not significant.

In addition, to investigate the influence of the mentioned clinical, endoscopic and functional variables on reflux esophagitis, a univariate analysis of clinical, endoscopic and functional parameters was performed, considering the presence of esophagitis as independent variable. We observed that hiatal hernia (*P* = 0.01), LES opening (*P* = 0.05), esophageal wave length (*P* = 0.01) and pathological reflux number (*P* = 0.05) were significantly related to the presence of esophagitis. However, *H. pylori* infection was not significantly related to the presence of reflux esophagitis.

## Discussion

The incidence of *H. pylori* infection in patients with GERD varies widely in literature from 30 to 90%, and is approximately of 35% in most series [12]. This heterogeneity between the studies may be due to the geographical location of the studies due to the difference in the prevalence of *H. pylori* in the Far East, North America and Western Europe [8]. It was suggested that *H. pylori* could contribute to GERD through different mechanisms: the development of antral gastritis which increases acid production, a decrease of LES pressure and impairment of gastric filling [10]. Nevertheless, the decreasing prevalence of *H. pylori* infection and related diseases (ulcer disease and gastric cancer) in Western countries has been paralleled by an increased incidence of gastroesophageal reflux and related complications. These epidemiological data do not support a causative role of *H. pylori* for reflux disease, but suggest a negative association [11].

Esophageal *H. pylori* infection is uncommon in patients with Barrett’s IM, dysplasia, or adenocarcinoma, and may be restricted to the non-intestinalized columnar epithelium. Gastric *H. pylori* infection may have a protective effect for the development of Barrett’s esophagus [13]; this data was also found in our study, although the small sample size should be emphasized. Other authors have even found a lower prevalence of *H. pylori* infection in patients with reflux symptoms and have suggested a 'protective' role of *H. pylori* infection against the development of esophageal diseases [14, 15]. Smout believed that pre-existing LES dysfunction and gastritis, and susceptibility to reflux, increased by a latent reflux, are probably causative factors contributing to esophageal diseases, rather than *H. pylori* infection [14]. Another possible explanation for the heterogeneous results regarding the relationship between GERD and *H. pylori* may be related to the variable effect of *H. pylori* on gastric acid.

According to Gisbert *et al*. [16], who found no influence of *H. pylori* infection either on pH-metric data or on endoscopic findings, in our trial out of 184 GERD patients, 46% were *H. pylori*-infected while 54% were *H. pylori*-negative; in addition we found no statistical difference regarding presence and severity of reflux esophagitis between patients with and without *H. pylori* infection. Contrary to our study, most trials base the correlation between *H. pylori* infection and GERD only on endoscopic observations; the endoscopic pattern of GERD patients is often normal, and 24-hour pH-monitoring revealed a high diagnostic accuracy for GERD [17, 18]. Actually, even if the role of acid secretion in esophageal lesions is undeniable, it does not seem increased in GERD patients [19, 20]. In the present study, we found no correlation between *H. pylori* infection and pH-metric data, and the mean value of the DeMeester score was similar in *H. pylori*-positive and -negative patients as found by Oberg *et al*. [21]. Further, the total time of acidification was similar in both groups as outlined by Oberg *et al*. [21], who did not find any correlation between *H. pylori* infection and esophageal exposure to acid, detected by 24 hours pH-metry, in patients with erosive esophagitis or Barrett's esophagus. Schwizer *et al*. [22] studied 70 patients with GERD treated with lansoprazole associated with clarithromycin and amoxicillin in patients with *H. pylori* infection. There was no difference in 24-hour pH values before and after *H. pylori* eradication, suggesting that *H. pylori* eradication did not affect distal esophageal acid exposure.

In our study, we found an unusual prevalence of hiatal hernia in contrast with some authors [23, 24]. We are not able to explain this data, however one theory is that it may be due to the excessive attention with which this condition was searched for. The incidence of patients with hiatal hernia and no *H. pylori* infection was higher than patients with *H. pylori* infection and hiatal hernia (91 versus 31; *P* <0.0001). Moreover, according to a past study [23] and this present one, the incidence of hiatal hernia did not associate with RE (*P* <0.0001). We found significant correlation between *H. pylori* infection and hiatal hernia, considered by some authors as a supporting element of GERD, and significantly associated with the development of esophagitis [23, 24].

Virulent strains of *H. pylori*, including those with a cytotoxin-associated gene named *cagA+*, have been reported to be associated with significant gastric inflammation [11]. *H. pylori* gastritis is accompanied by the release of nitric oxide, cytokines and prostaglandins that may impair afferent nerve function, reduce LES pressure and damage esophageal mucosa [25, 26]. However, in contrast to other authors [27, 28], in our trial LES pressure was similar in patients with and without *H. pylori* infection. Further, out of 184 GERD patients only 26% had LES pressure <14 mmHg, and LES opening (*P* = 0.05) and esophageal wave length (*P* = 0.01) were significantly related to esophagitis.

The relationship between *H. pylori* infection and gastric adenocarcinoma is also controversial. Some authors suggest an increased risk of gastric atrophy in *H. pylori*-positive patients treated with long-term proton pump inhibitor therapy. In a small subset of *H. pylori*-infected patients, chronic gastritis may lead to gastric atrophy and intestinal metaplasia, a potential precursor for gastric adenocarcinoma [29].

In a recent randomized controlled trial by Kuipers *et al*. [30], none of the *H. pylori*-positive GERD patients treated with anti-reflux surgery developed gastric atrophy, compared to 31% of patients treated with proton pump inhibitor therapy for an average of five years. Differently, in a long-term trial of GERD patients treated for years with omeprazole, there was an increase both in the severity of corpus gastritis and in gastric atrophy in *H. pylori*-positive patients [31]. Amongst the *H. pylori*-infected patients, atrophy was detected in 12% at baseline and 39% on follow-up.

Contrarily, it has been suggested that *H. pylori cagA+* may potentially protect against complications of GERD, such as Barrett's esophagus and dysplasia and adenocarcinoma [32, 33]. The *H. pylori* infection in patients with Barrett's esophagus has been reported in 12 to 60% cases [34–37]. A recent meta-analysis presented at Digestive Disease Week 2002 reported a negative association between the prevalence of *H. pylori* and *cagA* + *H. pylori* and reflux disease, Barrett's esophagus and esophageal adenocarcinoma [38].

## Conclusions

The exact association between *H. pylori* and reflux disease continues to be debated. Our clinical, endoscopic manometric and pH-metric data show a significant role of *H. pylori* infection neither in the development of GERD nor in the pathogenesis of reflux esophagitis. However, there are the works in the literature that analyze all the variables we considered. Nevertheless, current data do not provide sufficient evidence to define the relationship between *H. pylori* and GERD. We are fully in agreement with Wu *et al*. [19] in stating that a comparative study of *H. pylori* gastritis patterns between GERD patients and non-reflux controls is therefore mandatory.
